# Social determinants of health and treatment center affiliation: analysis from the sickle cell disease implementation consortium registry

**DOI:** 10.1186/s12913-024-10717-6

**Published:** 2024-03-06

**Authors:** Gustavo G. Mendez, Judith M. Nocek, Donald J. Brambilla, Sara Jacobs, Oladipo Cole, Julie Kanter, Jeffrey Glassberg, Kay L. Saving, Cathy L. Melvin, Robert W. Gibson, Marsha Treadwell, George L. Jackson, Allison A. King, Victor R. Gordeuk, Barbara Kroner, Lewis L. Hsu, Sickle Cell Disease Implementation Consortium

**Affiliations:** 1 University of Illinois Chicago, 840 S. Wood St., MC 856 Pediatrics, 60612 Chicago, IL USA; 2 RTI International, Research Triangle Park, USA; 3 Washington University, St. Louis, USA; 4 University of Alabama at Birmingham, Birmingham, USA; 5 Icahn School of Medicine at Mount Sinai, New York City, USA; 6 University of Illinois College of Medicine at Peoria, Peoria, USA; 7 Medical University of South Carolina, Charleston, USA; 8 Augusta University, Augusta, USA; 9 University of California San Francisco, San Francisco, USA; 10 Duke University, Durham, USA; 11 Durham Veterans Affairs Health Care System, Durham, USA; 12 University of Texas Southwestern Medical Center, Dallas, USA

**Keywords:** Sickle cell disease, Linkage to care, Distressed communities Index, Social determinants of health, SCD center affiliation

## Abstract

**Background:**

Adults with sickle cell disease (SCD) suffer early mortality and high morbidity. Many are not affiliated with SCD centers, defined as no ambulatory visit with a SCD specialist in 2 years. Negative social determinants of health (SDOH) can impair access to care. Hypothesis: Negative SDOH are more likely to be experienced by unaffiliated adults than adults who regularly receive expert SCD care.

**Methods:**

Cross-sectional analysis of the SCD Implementation Consortium (SCDIC) Registry, a convenience sample at 8 academic SCD centers in 2017–2019. A Distressed Communities Index (DCI) score was assigned to each registry member’s zip code. Insurance status and other barriers to care were self-reported. Most patients were enrolled in the clinic or hospital setting.

**Results:**

The SCDIC Registry enrolled 288 Unaffiliated and 2110 Affiliated SCD patients, ages 15-45y. The highest DCI quintile accounted for 39% of both Unaffiliated and Affiliated patients. Lack of health insurance was reported by 19% of Unaffiliated versus 7% of Affiliated patients. The most frequently selected barriers to care for both groups were “previous bad experience with the healthcare system” (40%) and “Worry about Cost” (17%). SCD co-morbidities had no straightforward trend of association with Unaffiliated status. The 8 sites’ results varied.

**Conclusion:**

The DCI economic measure of SDOH was not associated with Unaffiliated status of patients recruited in the health care delivery setting. SCDIC Registrants reside in more distressed communities than other Americans. Other SDOH themes of affordability and negative experiences might contribute to Unaffiliated status. Recruiting Unaffiliated SCD patients to care might benefit from systems adopting value-based patient-centered solutions.

## Background

Sickle cell disease (SCD) affects approximately 100,000 individuals in the United States, predominantly those of racial and ethnic minority groups [1]. It is characterized by intravascular sickling and intra- and extra-vascular destruction of oxygen-carrying red blood cells, resulting in chronic hemolytic anemia, severe pain crises, and end-organ damage. Beyond inpatient services for vaso-occlusive pain crises and other disease-related complications, effective SCD management requires access to a comprehensive range of preventive screenings and prophylactic treatments such as hydroxyurea or blood transfusions [2–4]. The previously high SCD child mortality rate has improved dramatically due to advances in disease treatment and management [1, 2]. Therefore, as more individuals with SCD survive to adulthood, greater emphasis should be placed on access to care and participation in long-term comprehensive management of disease complications.

Although there is no accurate count of SCD patients in the USA [5], evidence suggests that comprehensive care with a sickle cell hematologist is only reaching 2/3 of children with SCD [6], and less than half of adults with SCD [7]. Social issues around access to quality SCD care also persist in teenage patients and adults. Adolescents and young adults have low rates of ambulatory clinic attendance compared to Emergency Department visits for SCD or pain [8]. Individuals (especially young adults) with SCD might not receive necessary comprehensive care for many reasons: the scarcity of adult-oriented providers with specialization in SCD, lack of insurance, and loss of appropriate care due to transition to adulthood [8–14]. As a result of these systemic barriers, affected individuals are not receiving disease-specific care such as hydroxyurea [5, 15, 16], potentially resulting in greater morbidity and contributing to early mortality.

This at-risk group of individuals, termed *Unaffiliated patients* [17, 18] often do not receive necessary preventative care, disease-modifying therapy, education in SCD management, or the opportunity to participate in research or advocacy. In the United States, identifying such Unaffiliated persons is difficult because there is no national SCD registry or data capture system [5, 19]. Also, administrative datasets fail to capture Unaffiliated patients if they are not seen routinely or if they have been misdiagnosed [19].

Although SCD is a genetic disease, overall health can be negatively influenced by lower socioeconomic status, lack of insurance, racial discrimination, and other adverse social determinants of health (SDOH) [10, 11, 14, 20–27]. A recent theoretical review subsumes SDOH under an expanded concept of diversity [28] and explains how diversity plays an important role in health inequality [29]. The review article points to three theoretical approaches to conceptualizing SDOH. The first approach examines diversity on the psychological level; SDOH can be represented by diverse real or perceived perceptions of unequal treatment in healthcare. This can lead to stress and poor health [14, 26]. The second approach, on a socio-economic level, places an emphasis on economic diversity in healthcare treatment [30]. Finally, a multilevel conceptualization of SDOH considers the combination of biological, social, economic, and historical factors [30, 31]. 

Most of the research on the relationship between SDOH and access to higher acute care utilization has been conducted among children [4, 6, 11]. Relatively little is known about the contribution of SDOH to adult affiliation and attendance at a SCD treatment center.

**Fig. 1:**
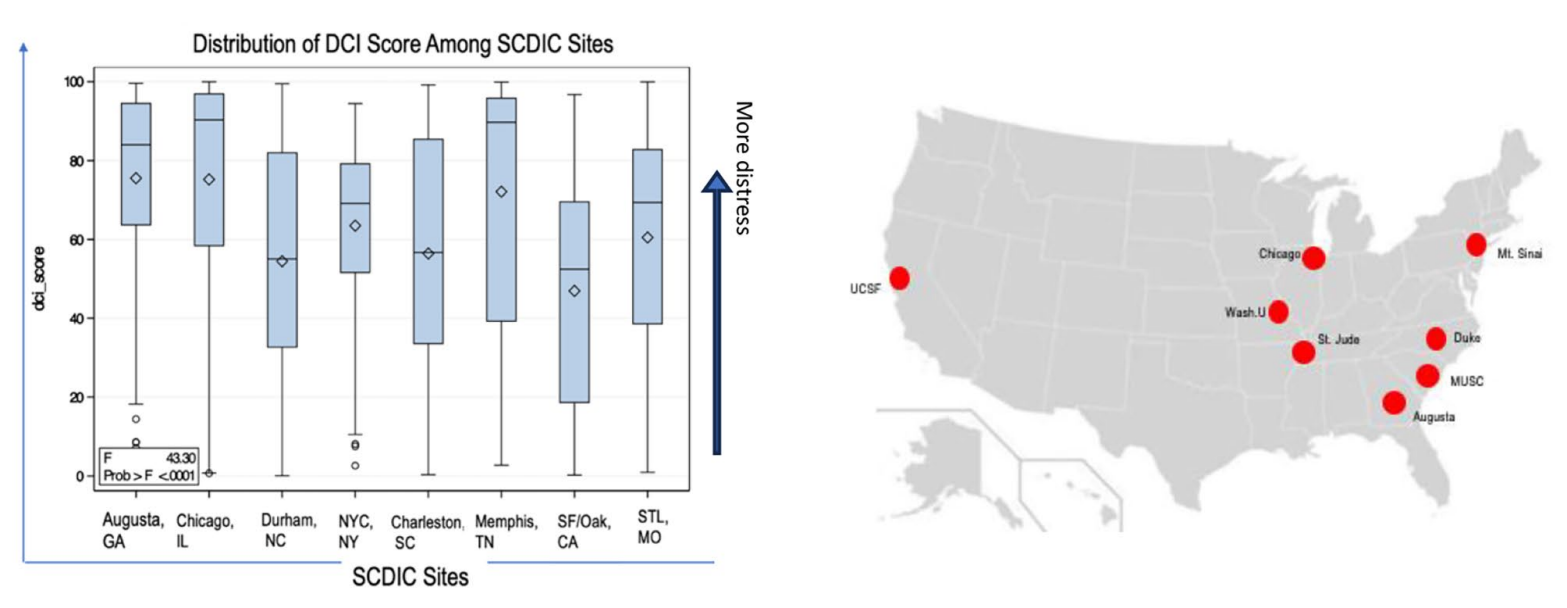
SCDIC sites and distribution of DCI scores for each site. Left panel: Distributions of DCI scores shown as box and whisker plots The vertical line for each box shows the interquartile range (IQR), the horizontal line is the median, and the diamond is the mean. Right panel: locations of 8 sites in SCDIC

To assess utilization of comprehensive care for adolescents and young adults with SCD the National Heart, Lung and Blood Institute (NHLBI) funded the Sickle Cell Disease Implementation Consortium (SCDIC) comprised of eight comprehensive sickle cell centers in the United States [17, 32, 33]. These eight sites represent a large geographic area of the United States, including coastal, midwestern and southern regions with urban, suburban, and rural populations with SCD (see Fig. 1).

The purpose of our study was to explore whether individual and SDOH differences were associated with SCD treatment center affiliation status in the SCDIC Registry for patients aged 15 to 45. We also explored evidence for alternative factors in Unaffiliation: that individuals with milder disease course might perceive low value in ambulatory SCD preventive care, or that previous negative experiences might dissuade them from seeking such care.

## Methods

The SCDIC created a patient registry combining disease and demographic information from the electronic medical record with data acquired through quantitative and qualitative patient surveys [9, 17, 32–35]. The eight-site SCDIC Registry enrolled 2,400 individuals with SCD ages 15–45, 300 from each site, using a convenience sample enrollment strategy. Inclusion and exclusion criteria were: (i) had received a confirmed diagnosis of SCD of any genotype, (ii) lived in the geographic region of 1 of the 8 SCDIC sites, (iii) between 15 and 45 years of age. For the most part, patients were enrolled in the clinic or hospital setting during a sickle cell-related outpatient or inpatient visit. A small honorarium or gift card was offered in compensation for the time invested in completing the questionnaire. Data from questionnaires administered at annual Registry follow-ups are presented elsewhere [9, 35]. The current study was a cross-sectional analysis of the SCDIC Registry. No permission was needed for access to the data, because this study was conducted by the investigators and sites that established the Registry. In December 2023, Registry data were transferred to the NHLBI BioLINCC data repository, where the data are available for sharing with investigators outside of the SCDIC.

### Affiliation status

The definition used for Affiliation status was developed by a consensus of the SCD investigators and patient stakeholders in the SCDIC in 2017: Unaffiliated patients were those who did not have routine, scheduled ambulatory care with a sickle cell expert for the 24 months preceding enrollment into the Registry. Affiliation status for each patient was determined using chart review by SCDIC personnel and by self-report with the question “What type of healthcare professional has been providing the majority of care for your sickle cell disease in the past 2 years?” [17]. A subsequent Delphi consensus after Registry completion set a tighter timeframe definition of 12 months for Unaffiliation [18], but data were not collected on that definition during the SCDIC Registry. In a few cases, SCDIC personnel had no access to medical records but found the self-reported Unaffiliated status likely to be valid because the individual resided where there was no sickle cell expert. Those who met the above criteria were coded as Unaffiliated while the rest of the patients were coded as Affiliated.

### Registry data

Mental/behavioral health comorbidities were limited to the diagnostic categories of depression and anxiety by physician note or ICD10 codes; all other mental/behavioral health diagnoses were rarely reported and were omitted from this analysis. Although many SCD complications were recorded in the Registry baseline data, some SCD complications less subject to reporting bias or inaccurate coding included: ischemic stroke, hemorrhagic stroke, acute chest syndrome, and chronic pain. The diagnosis of “left ventricular dysfunction” used in this research, could be applied to the clinically unimportant left ventricular hypertrophy that compensates for anemia or the clinically serious left ventricular diastolic dysfunction. Data collection forms have been published elsewhere [17]. 

### Distressed communities index

For privacy reasons in rare disease, the SCDIC Registry collected zip codes rather than residence address or census tract. The Distressed Communities Index (DCI) has been used to analyze socioeconomic factors in SCD by zip code [36, 37]. DCI for the zip codes of enrolled patients was obtained from the Economics Innovation Group website [38], The DCI used in this research is a combination of 7 components collected as part of the US Census Bureau American Community Survey, as a metric for the economic disparities between US zip codes: (1) Percent of adults without a high school diploma, (2) Poverty rate, (3) Percent of adults not working, (4) Housing vacancy rate, (5) Median household income, (6) Change in employment, and (7) Change in the number of business establishments. DCI scores were calculated by the Economics Innovation Group by ranking US zip codes on each of the 7 measures, calculating the average rank for each zip code, and scaling average ranks to range from possible scores of zero to 100. The median scaled rank was set to 50. Zip codes in the highest quintile (DCI scores 81–100) were categorized as “distressed communities”. Those in the next quintile (DCI scores 61–80) were categorized as “at-risk communities.” DCIs for year 2018 were obtained from the website by entering the residence zip code self-reported by individuals in the SCDIC Registry.

### Data analysis

Data were entered in a REDCap database for centralized data capture and management at RTI International. Chi square tests were employed to evaluate the associations between Affiliation status and individual categorical predictors, as well as variation in the frequencies of the five quintiles of DCI among the sites. Logistic regression was used to evaluate multiple predictors of Affiliation status in the same model. DCI scores were compared among sites using one-way analysis of variance (ANOVA) with Tukey’s studentized range test for pairwise comparisons.

## Results

### Site characteristics

Sites were in Augusta GA (Augusta University), Chicago IL (University of Illinois), Durham NC (Duke University), NYC NY (Mt. Sinai/Icahn), Charleston SC (MUSC), Memphis TN (St. Jude), San Francisco/Oakland CA (Benioff), and Saint Louis MO (Washington University). Four sites were in states that adopted Medicaid expansion (CA, IL, MO, NY) and four were in states that did not (GA, NC, SC, TN). The dominant source of health insurance for Americans with SCD is Medicaid [39]. Medicaid is a government-funded, comprehensive health insurance program that covers children, pregnant women, low-income adults, and people with disabilities. States with “Medicaid expansion” set eligibility on low-income level alone. In states that did not adopt “Medicaid expansion” limit, eligibility was based on household size, disability, family status, and other factors in addition to income level.

### Distressed community score

Figure 1 also shows box and whisker plots for the DCI score for each site. Of the 2,392 patients in the entire registry, 486 (20%) resided in the at-risk quintile (DCI 61 to 80) and 926 (39%) resided in the distressed quintile (DCI 81 to 100). An ANOVA of DCI scores across the 8 sites showed statistically significant variation (*p* < 0.001). Based on Tukey’s studentized range test, Augusta, Chicago, and St. Jude clustered with the highest mean DCIs (72.1–75.5). UCSF was alone with the lowest mean DCI (46.9). The DCI means for the other four sites fell between 54.5 and 63.5. A comparison of the percentages in the highest DCI quintile produced similar results; 53%, 59% and 65% of subjects were in the distressed quintile in Augusta, Chicago, and St. Jude. Only 13% of subjects at UCSF were in the distressed quintile. The percentages in the distressed quintile ranged from 22.1 (Mt Sinai) to 33.5 (MUSC) in the other four communities. Table 1 shows the percent of registry patients in the highest DCI quintile by Affiliation Status.

**Table 1:** SCDIC patient registry DCI quintile by affiliation status

Distress level	DCI quintile vs. Affiliation status			
	Count AFF	AFF %	Count UNAFF	UNAFF %
*Prosperous DCI 0–20*	274	13.19	26	9.22
*Comfortable DCI 20–40*	251	12.08	31	10.99
*Mid-tier DCI 40–60*	334	16.07	49	17.38
*At risk DCI 60–80*	411	19.78	67	23.76
*Distressed DCI 80–100*	808	38.88	109	38.65
*Total*	*n* = 2078	100%	*n* = 282	100%

There was no statistically significant difference between the two Affiliation groups at any site (χ^2^_(4)=_0.660, ns).

### Population characteristics

**Fig. 2:**
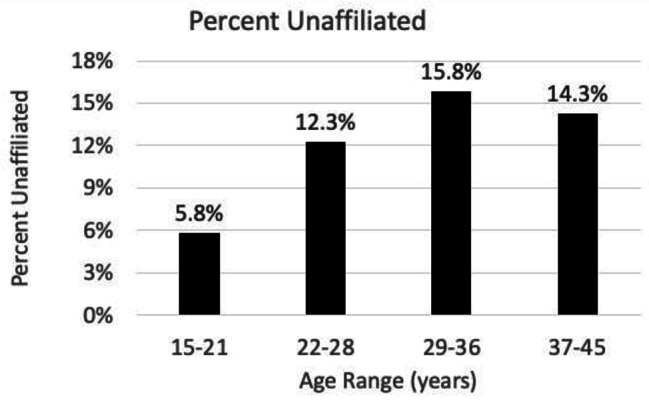
Percent unaffiliated by age category

Table 2 shows that gender did not differ significantly across Affiliation groups. The frequencies in the age categories, also presented in Table 2, show different affiliation status patterns by age group ( χ^2^_(3)_ = 31.71, *p* < 0.0001). As shown in Fig. 2, the Affiliation status appears to be quadratically related to age. The frequency of Unaffiliation rose with age from adolescence until the 29-to-36-year age category, then Unaffiliation decreased in the middle-aged adults.

**Table 2:** Patient demographics and SCD genotype by affiliation status

	Affiliated		Unaffiliated		Total	
	**Freq.**	**Column Percent**	**Freq.**	**Column Percent**	**Freq.**	**Column Percent**
**Gender**						
Female	1192	56.5%	166	57.6%	1358	56.6%
Male	918	43.5%	122	42.4%	1040	43.4%
**Totals**	**2110**		**288**		**2398**	
**Age Group Row Percent**	**Freq.**	**Row Percent**	**Freq.**	**Row Percent**	**Freq.**	
15–21	532	94.2%	33	5.8%	565	
22–28	643	87.7%	90	12.3%	733	
29–36	607	84.2%	114	15.8%	721	
37–45	300	85.7%	50	14.3%	350	
**Totals**	**2082**	**87.9%**	**287**	12.1%	**2369**	
**Genotype**	**Freq.**	**Column Percent**	**Freq.**	**Column Percent**	**Freq.**	**Column Percent**
Hb SS	1468	69.6%	184	65.2%	1652	69.1%
Hb SC	423	20.1%	70	24.8%	493	20.6%
Hb S beta + thalassemia	114	5.4%	16	5.7%	130	5.4%
Hb S beta0 thalassemia	82	3.9%	9	3.2%	91	3.8%
Hb Other	21	1.0%	3	1.1%	24	1.0%
**Totals**	**2108**		**282**		**2390**	

The distribution of SCD genotypes in the SCDIC data, shown in Table 2, is similar to the genotype distributions seen across the United States [40]. The trend of a slightly larger percentage of sickle cell disease SC type among the Unaffiliated patients compared to the Affiliated (25% vs. 20%] was not statistically significant.

### Income and insurance

**Fig. 3:**
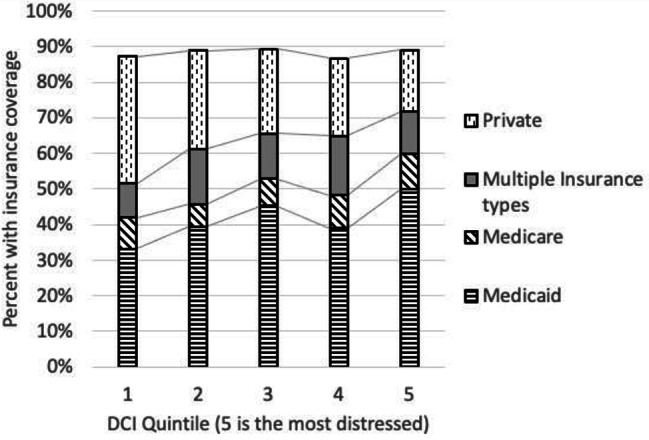
Insurance type by DCI quintile

Seventy-six percent of the sample reported a household income of $50,000 or less. There was no statistically significant association between Affiliation status and self-reported income (χ^2^_(4)_ = 0.386, ns). An analysis of the relationship between insurance type and Affiliation status shows that a larger proportion of Unaffiliated patients are uninsured (19%) compared to Affiliated (7%). Fewer Unaffiliated registry participants relied on Medicaid (34%) compared to those who were Affiliated (45%). Insurance type did vary by DCI quintile. As community distress increased, a larger percentage of patients were covered by Medicaid and a smaller percentage were covered by private insurance (see Fig. 3).

### Hydroxyurea treatment

Hydroxyurea is strongly recommended in guidelines for SCD as a disease-modifying agent that reduces the severity and frequency of SCD complications [15, 41, 42]. 

Analysis of hydroxyurea prescription status, using data that was obtained from patients’ medical records, showed a small but not statistically significant difference by Affiliation Status. Of the 1,926 Affiliated patients 1,147 (58.9%) were current users, 304 (15.6%) were former users and 495 (25.4%) had never used HU. Of the 221 Unaffiliated patients, 99 (44.8%) were current users, 43 (19.5%) were former users and 79 (35.8%) had never used HU. The association between level of HU use and Affiliation status was not statistically significant (χ^2^_(2)_ = 0.88, ns).

### Co-morbid conditions

The next analysis examined the relationship between Affiliation status and SCD-related complications. First, logistic regression was used to test associations between Affiliation Status and nine common SCD-related chronic conditions. Second, logistic regression was used to test associations between Affiliation Status and SCD acute symptoms that may show up when a SCD patient goes to an ED.

Patients were more likely to be in the Affiliated group if they had chronic co-morbidities of priapism, asthma, gallstones and cholecystitis, anxiety, and depression (Table 3), when controlling for patient age group. Conversely, patients with left ventricular dysfunction were more likely to be in the Unaffiliated group. The association between avascular necrosis and Affiliation status was statistically significant (*p* = 0.049) in the full model and but was non-significant in a backwards elimination model (not shown). All the other above-mentioned chronic comorbidities survived the backwards elimination.

Holding constant these comorbidities, patients in the 15 to 18 year age group were significantly more likely to be Affiliated with a SCD comprehensive care center compared to those in the 38 to 45 year old age group while those in the 29 to 37 year age group were more likely to be in the Unaffiliated compared to those in the 38 to 45 year age group.

**Table 3:** Logistic regression of the probability of being unaffiliated when all 9 predictors are in the model

Variables		DF	Estimate	SE	Chi-Square	*P*
Intercept		1	-1.7003	0.1078	248.7761	< 0.0001
Age	15–21	1	-0.8792	0.1588	30.6522	< 0.0001
	22–28	1	0.1235	0.1145	1.1634	0.2808
	29–37	1	0.3859	0.1130	11.6658	0.0006
	38–45 (reference group)	--	0	--	--	--
Complications	Avascular necrosis	1	-0.3294	0.1677	3.8597	0.0495
	Priapism	1	-0.6275	0.2729	5.2856	0.0215
	Left ventricular dysfunction	1	0.7208	0.3265	4.8725	0.0273
	Asthma	1	0.0279	0.1707	0.0266	0.8703
	Gallstones/ cholecystitis	1	-0.3959	0.1453	7.4216	0.0064
	Hypersplenism	1	0.0997	0.4857	0.0422	0.8373
	Skin Ulcers	1	-0.6393	0.4359	2.1506	0.1425
	Anxiety	1	-1.0038	0.3355	8.9526	0.0028
	Depression	1	-0.5409	0.2296	5.5525	0.0185

A second analysis examined the relationship between acute conditions likely to be diagnosed when a patient with SCD comes to a hospital ED [8, 11, 43, 44] and Affiliation status. Results from a logistic regression analysis are shown in Table 4. Controlling for age group, acute chest syndrome, splenectomy, and chronic refractory pain were independently and statistically significantly associated with being in the Affiliated group. As in the analysis presented in Tables 3 and 15 to 18 and 19 to 21 year old patients remained significantly more likely to be in the Affiliated group compared those in the 38 to 45 year age group but the difference with the 29 to 45 year age group category was not significantly associated with Unaffiliation as it was in the previous analysis.

**Table 4:** Logistic regression of the probability of being unaffiliated when all 8 predictors are in the model

Parameter		DF	Estimate	SE	Wald Chi-Square	*P*
Intercept		1	-1.8309	0.1253	213.6523	< 0.0001
Age Group	15–21	**1**	**-0.5106**	**0.1760**	**8.4123**	**0.0037**
	22–28	1	-0.0032	0.1440	0.0005	0.9821
	29–37	1	0.2157	0.1357	2.5264	0.1120
	38–45 (reference group)	--	0	--	--	--
Symptoms	Dactylitis	1	-13.8607	407.1	0.0012	0.9728
	Ischemic stroke	1	0.1965	0.3567	0.3035	0.5817
	Hemorrhagic stroke	1	-14.3177	747.4	0.0004	0.9847
	Intracranial bleed	1	0.4405	1.1293	0.1522	0.6965
	Acute chest syndrome	**1**	**-0.3763**	**0.1749**	**4.6304**	**0.0314**
	Splenic sequestration	1	-0.0095	0.3697	0.0007	0.9796
	Splenectomy	**1**	**-0.6695**	**0.3471**	**3.7198**	**0.0538**
	Chronic refractory pain	**1**	**-0.8597**	**0.2525**	**11.5917**	**0.0007**

### Health system barriers to health care

Registrants were presented with a survey questionnaire asking about barriers to comprehensive care. The initial question “During the past 12 months, was there any time when you didn’t get the medical care you needed or had delays in getting the care you needed?” was affirmed by 37.5% of Unaffiliated compared to 30.6% of Affiliated (χ^2^_(1)_ = 5.53, *p* = 0.019). Follow-up questions about specific barriers did not show significant differences between Affiliated and Unaffiliated patients. The most frequently endorsed barrier to care was “You had a previous bad experience with the health care system” - selected by 45% of Unaffiliated and 43% of Affiliated. Interestingly, several other barriers were selected less often by Unaffiliated than Affiliated: “You couldn’t get an appointment soon enough” (25% vs. 34.1%), “You couldn’t get there when the doctor’s office or clinic was open” (12% vs 16%), “It takes too long to get to the doctor’s office from your house or work” (10.2% vs. 12.9%), and “You couldn’t get through on the telephone” (13% vs. 18.4%). Some barriers vary significantly between sites. “The doctor or hospital wouldn’t accept your health insurance” was selected by 19.5% at USCF and 18% at Chicago but only 3.5% at Duke and 2.3% at MUSC. “You couldn’t get through on the telephone” was selected by 23% at Duke but only 2.1% at St. Jude and 4.1% at Augusta.

## Discussion

Other studies have associated negative social determinants of health, at either the neighborhood level or the individual level, with lower utilization of ambulatory care in SCD [45, 46]. Those studies of SDOH emphasized pediatric SCD populations in contrast to the adolescents and young adults in the SCDIC Registry. The SCDIC Registry findings that neighborhood DCI was not correlated with Unaffiliation resemble pediatric SCD care in the Detroit area, in which low SCD stroke screening rates did not correlate with neighborhood conditions, and showed SCD children have a high level of socioeconomic disadvantage [47]. Zipcode of residence was in most distressed quintile of DCI for 38.88% of Affiliated and 38.65% of Unaffiliated SCDIC Registry participants, which is similar to national data showing residence in the most distressed quintile of DCI for 35.3% of Black Americans in 2020 [38].

### Health insurance coverage

Access to health insurance is a major contributor to overall health for the general population and an important social determinant of health in socioeconomically disadvantaged communities. Lack of health insurance is a well-known barrier to care in the United States. Study results suggest differences in insurance coverage for Unaffiliated vs. Affiliated patients with SCD. Affiliated patients were more likely to have Medicaid. Those classified as Unaffiliated reported less reliance on Medicaid (34%), the same reliance on private insurance (23%), and a higher percentage uninsured (19%). Additional analysis showed that the type of insurance coverage varied by community distress (Fig. 3). These differences may highlight a potential barrier to continuous expert care for Unaffiliated SCD patients. The sample size was insufficient to analyze whether insurance coverage was associated with Affiliation within each quintile. Kayle and colleagues [39] reported that Medicaid expansion in California was associated with a complicated impact on patients with SCD: desirable trends in hydroxyurea and ED usage, but reduction in Medicaid enrollment over a three-year period. They did not analyze ambulatory care or Affiliation with SCD centers [39].

To better understand the impact of insurance on health care Affiliation status, it is important to understand that Medicaid access to healthcare does not remove costs and income inequality as barriers to healthcare access [48]. For instance, in 2018 the average American family spent $8,200 (or 11% of the family income per year) on health care premiums and out-of-pocket costs for items such as office visit copays, prescription drugs, and surprise or “out of coverage” medical bills [25]. One analysis of the financial impact of SCD finds 4-fold higher out-of-pocket costs than in matched controls, which can be overwhelming to households that are already facing economic difficulties [49]. Further consideration can also be placed on the 87 million American adults (aged 19 to 64) who are reported by the Commonwealth Fund as *underinsured* [50]. Underinsured people have health insurance coverage that leaves them with high out-of-pocket cost relative to income and 18% of those 87 million are African Americans.

For these reasons, Affiliation status to a consistent SCD expert may be linked to healthcare policy and access. Another perspective might also be the “value proposition” for the individual with SCD in the context of value-based health [5, 51, 52]; the value of seeing the sickle cell expert must show that the benefits are worth the costs. There is a personal financial cost to seeking health care: out-of-pocket health expenses, transportation to care, costs of absence from work or school [49]. Other costs may be emotional like returning to a health system where you had a bad experience or difficulty [23, 53]. 

In the survey about health system barriers to care, responses could be categorized in the framework of a value proposition. Bad experiences appeared to be more commonly endorsed by Unaffiliated patients compare to Affiliated “You had a previous bad experience with the health care system” (45% vs. 43%) and “Issues with ED” (14.8% vs. 10%). Cost concerns were also more commonly endorsed by Unaffiliated “Worry about the Cost” (24% vs. 17.3%), and “Lack of Health insurance” (4.6% vs. 2.1%). Thus, health system barriers are a major problem for both Affiliated and Unaffiliated patients, with fairly small differences between these groups. The barriers to care are very heterogenous between sites as well as between individuals. A health system could potentially reduce some barriers like phone access and time to appointment [9, 35]. Building a trusted relationship might overcome previous bad experiences. Other barriers are caused by the American health insurance situation such as: worry about cost, not accepting your insurance, a health plan wouldn’t cover the treatment, and lack of insurance in a small percentage of this sample.

In summary, these DCI data are similar to pediatric SDOH data in SCD and indicate that adults with SCD have greater economic hardship than the general population [54, 55]. DCI does not capture the contributing factors in the Affiliation status of adults with SCD in the SCDIC Registry. According to these results, Unaffiliation depends on age, consistent with poor transitions from adolescent to adult care. Other measures of SDOH might need to be emphasized, such as high out-of-pocket costs, that might require very specific questions to be asked [49, 56]. Inadequate health insurance coverage is a major SDOH barrier to care, especially in the context of high rates of poverty and high rates of underemployment associated with SCD [5]. Previous bad experiences and difficult access to appointments might also contribute to Unaffiliated status. Therefore, reasoning from a “consumer value equation” perspective, we considered the alternative hypothesis that SCD patients suffering fewer medical complications might be Unaffiliated because they have lower motivation to overcome SDOH and seek regular expert SCD care.

### Medical complications and comorbidities

Unsurprisingly, affiliated patients are likely to be sicker patients with SCD in the SCDIC registry. We expected mental health problems to cause difficulty navigating an appointment with the SCD specialist and to be associated with Unaffiliated status. However, patients with stroke, depression, and anxiety were less likely to be Unaffiliated. Depression and anxiety have high prevalence in SCD and have complex interactions with chronic pain, health-related quality of life, and high utilization of ED and hospitalizations [43]. It is possible that ascertainment bias led to underdiagnosis of mental health problems in the Unaffiliated patients such that Unaffiliated patients with fewer mental health problems did not seek professional health.

We expected frequent pain or ACS to correlate with acute hospital usage but not necessarily ambulatory care [49]. This analysis showed that chronic pain and ACS were associated with the lower likelihood of Unaffiliated status, suggesting that these adults are getting expert ambulatory care. They might have been more motivated for ambulatory care to get hydroxyurea treatment or pulmonary care. They might also have been more visible to the medical team and have linkage to ambulatory care provided upon hospital discharge.

We expected major organ damage like renal failure or pulmonary hypertension to cause people to seek ambulatory care with multiple specialists [56], which complicates the categorization of Affiliated vs. Unaffiliated status, and will be reserved for a future report. Unexpectedly, those with the complication of left ventricular dysfunction were significantly more likely to be Unaffiliated. It is possible that ascertainment bias by non-experts led to misdiagnosis through misinterpretation of left ventricular compensation for anemia in the Unaffiliated patients. It is also possible that symptoms of left ventricular dysfunction are more insidious in onset and could be thought to be secondary to chronic anemia, so that individuals are not triggered to seek medical care.

Hydroxyurea is recommended strongly in guidelines for SCD as a disease-modifying agent that reduces the severity and frequency of SCD complications [41]. Patients who are Unaffiliated with the SCD expert might not find a physician willing to prescribe hydroxyurea [15, 57]. Fewer Unaffiliated patients are currently taking Hydroxyurea (45%) compared to those Affiliated (59%). In addition, more Unaffiliated patients had never taken hydroxyurea (36%) compared to those Affiliated (25%).

In summary, the medical history had both expected and unexpected relationships to Unaffiliated status. These differences could reflect the different healthcare landscape that a working-class SCD patient may face when compared to the poorer SCD patient. The trends in SCD complications, hydroxyurea usage, and mental/behavioral health are consistent with the alternative hypothesis: Unaffiliated patients could be those less severely affected by SCD. The non-significant trend toward difference in genotype may reflect the likelihood that more severe SCD will drive patients into long-term expert care, whereas less severe disease and less acute symptoms can allow patients longer periods of time without expert care.

The strengths of these data are the large sample size with combined clinical chart abstraction and self-report that are more detailed than an administrative dataset can provide. Another strength is that these data are from 2018, which is in the current era of access to care, compared to previous data prior to the Affordable Care Act. One limitation is that the Unaffiliated status was defined in 2017 for the Registry as 2 years without a scheduled ambulatory encounter in the sickle cell specialist center, prior to the Delphi consensus that defined Unaffiliated as 1 year without seeing a sickle cell specialist [18]. The data capture for this study used a binary measure and an entirely new chart abstraction which is not feasible for 2400 subjects at this point. However, the definition used in this study is a longer period of Unaffiliation, and thus could be regarded as representing individuals who are more entrenched in the Unaffiliated status. Another major limitation of these data as descriptors of the Unaffiliated group is the sampling strategy of the SCDIC registry. The registry is comprised primarily of a convenience sample of those who were already close enough to the academic SCD center to enroll– this strategy leaves out those who cannot or will not contact the SCD center’s hematologist. Outreach through SCD community-based organizations did enroll a few Unaffiliated patients, but outreach recruitment was limited by the timeline of the Registry. A recent estimate from commercial and Medicaid administrative claims databases found that the proportion of SCD patients who saw a hematologist in the prior year was 39–47% in private health insurance and 2–15% in Medicaid insurance [7], which implies that 53–85% of SCD patients are Unaffiliated [7]. The data missing on this large Unaffiliated population might not be represented by the individuals interviewed in this report. Other measures of social deprivation and SDOH exist, although skewed toward pediatric metrics [45, 58–60]. Future studies of Unaffiliated patients will need to devote large resources to finding the individuals who might have no contact with the SCD center and might be trying to avoid being found due to previous bad experiences.

### Implications for finding unaffiliated patients and linking to care

These results demonstrate that, as with many health disparities, the needs of Unaffiliated patients with SCD are heterogenous and diverse. Just as in other applications of value-based, patient-centered care, the medical system might need to invest more effort in showing individuals with SCD the benefits of health care. To recruit the Unaffiliated patients back to ambulatory care, a one-size-fits-all solution is unlikely to be successful. It is necessary to identify the individual needs of Unaffiliated patients and engage them to optimize their SCD care. These individual differences and needs also occur within the context of communities, healthcare systems, and policies that vary by state and region. Although we did not see an association between the specific measure of DCI and being Unaffiliated, efforts to implement programs aimed at connecting Unaffiliated patients with care likely still need to account for the ways in which individuals are impacted by the environments in which they live and seek care, including different aspects of care that may be impacted and the process of implementation [45, 61–64]. 

The problem of Unaffiliation is not unique to SCD and has been identified in other chronic illnesses. In HIV/AIDS, depression, and hepatitis C, the reasons for non-affiliation have been reconceptualized from *a patient-focused* view (e.g., what are the characteristics of patients that make them not seek evidence-based health care?), to addressing *systems-based* issues that may hinder affiliation [7, 56, 57, 62, 65–71]. This perspective would place responsibility for the problem of non-affiliation on the healthcare *system*, not on the*individual* disconnected from care. We note that the terminology differs in each field: “linkage and retention” in the HIV/AIDS field [69–71], “re-engage in treatment” in mental health [72], “adherence with preventive care” in health maintenance organizations [73], “adherence” in cancer [74] and cystic fibrosis [75], “compliance” or “lost to follow up” in other fields [76].

A toolkit has been developed by SCDIC to address the heterogeneous ways in which individuals become unaffiliated from, or were never affiliated with, the healthcare system.

The first set of strategies would focus on ***finding the Unaffiliated patients*** with SCD using three general pathways: (1) community-based pathway, (2) hospital-based pathway, and (3) SCD surveillance pathway. The *community-based recruitment* strategies are designed to reflect a sensitivity to the underlying reasons why individuals with SCD are Unaffiliated in the first place, including potential mistrust of the healthcare system, and to incorporate known information about turning points that contribute to why people do not affiliate. The community-based pathway encourages novel community partnerships for patient engagement. The *hospital-based pathway* refers to identifying patients in acute care settings who are not in SCD specialty care. Finally, the *SCD surveillance pathway* can draw upon the CDC Sickle Cell Data Collection (SCDC) Program in four of seven states to identify community hospitals without SCD specialists who are seeing large numbers of affected individuals.

After the Unaffiliated patients are found, the next stage would be ***linking and retaining them to care***. Identifying Unaffiliated patients is a necessary but insufficient step in the process of affiliation. In HIV/AIDS programs, a systemic approach to intervening in the problem of non- affiliation is the Linkage Coordinator (LC) [71]. The LC will receive contact information for the Unaffiliated patients identified through the pathways and will be responsible for personally connecting with patients, addressing barriers that previously disconnected them from care, providing SCD education, and connecting patients to a SCD specialist. The LC will use a patient-centered approach to bridge the gap between the barriers to affiliation and quality care. The LC can use techniques like care coordination, motivational interviewing, and personalized reminder calls. An LC with a “high touch” approach, building a personal connection with the patient based on a shared background, can be particularly helpful for underserved minorities who have been mistreated by institutional racism [77]. 

LCs have not been formally used or studied with the SCD population. However, patient navigators and SCD adolescent peer-patient advocates have some overlap with the roles of the LC [78]. Community health worker training is available through the Sickle Cell Disease Association of America and other organizations to provide some of the background necessary for a SCD LC. Community advisors can help evaluate the process, interventions, and outcomes.

Finally, ***implementing change at large scale*** to improve the healthcare system to find and link the Unaffiliated means addressing the systemic problems that contributed to some individuals becoming Unaffiliated [65, 74, 79, 80]. Implementation scientists usually assess needs and develop interventions for *known* populations [65, 74, 79, 80]. In the problem of Unaffiliated SCD patients, interventions must also reach *unknown* members of the target population. Across diseases, some subgroups are always missing from calculations of reach. Strategies derived from data only on known populations will not be generalizable to these unknown members [58, 65, 74, 77, 79, 80]. Scaling up the SCD interventions for Unaffiliated patients requires understanding these individuals. Implementation health systems that can increase the number of SCD experts will help scale up the capacity for compassionate care and taking the time to understand the Unaffiliated patient.

## Conclusions

Overall, these results indicate the heterogeneity of the Unaffiliated group. The Distressed Communities Index is a simple zip code economic measure of SDOH but showed no utility in predicting Unaffiliated status in the SCDIC Registry. DCI is an ecological measure of SDOH and does not assess SDOH factors at the household or individual level. SDOH measures at the individual level could be more important for Unaffiliated status. Individuals report multiple barriers to care besides insurance status, but sites differ in their populations and their barriers. Motivation to seek expert care might correlate with the greater severity of SCD complications and mental/behavioral health co-morbidities. Another perspective might be that affiliation depends on showing patients a favorable “value proposition” of benefits vs. costs, implying that the health system might use a marketing approach to demonstrate this “value proposition” for seeing the sickle cell expert. The health system can also address emotional costs to patients like returning to care within a health system where they had a bad experience. This heterogeneity implies that reducing the number of Unaffiliated patients with SCD will not have a “one-size fits all” solution.

Continuity of care is an important topic for future study, as well as methods for linking patients to continuity of care. The value of Linkage Coordinator has been established in other fields such as HIV [71, 81–83]. Motivational encouragement and care coordination to overcome individual problems of access to care, coupled with adaptation of strategies for the local needs at each site are necessary. Personal bonding with a linkage specialist could help overcome the barrier of a previous bad experience with the healthcare system, making implementation of a Linkage Coordinator program for SCD Unaffiliated patients one very feasible solution to Unaffiliation.

## Data Availability

SCDIC Registry data became available to researchers in 2024 through the NHLBI Biologic Specimen and Data Repository Information Coordinating Center (BioLINCC) at https://biolincc.nhlbi.nih.gov/home/.

## Abbreviations

BioLINCC: Biologic Specimen and Data Repository Information Coordinating Center
DCI: Distressed Communities Index
ICD10: International Classification of Diseases 10
IRB: Institutional Review Board
NHLBI: National Heart, Lung, and Blood Institute
SCD: Sickle cell disease
SCDIC: Sickle Cell Disease Implementation Consortium
SDOH: Social determinants of health
